# 经典型霍奇金淋巴瘤的治疗挑战与优化

**DOI:** 10.3760/cma.j.cn121090-20240906-00338

**Published:** 2025-04

**Authors:** 坚青 糜, 域 方, 文嫣 虞, 清清 蔡

**Affiliations:** 1 上海血液学研究所，医学基因组学国家重点实验室，国家转化医学中心（上海），上海交通大学医学院附属瑞金医院，上海 200025 Shanghai Institute of Hematology, State Key Laboratory of Medical Genomics, National Research Center for Translational Medicine at Shanghai, Ruijin Hospital Affiliated to Shanghai Jiao Tong University School of Medicine, Shanghai 200025 China; 2 华南肿瘤学国家重点实验室，肿瘤医学协同创新中心，中山大学肿瘤防治中心，广州 510060 Department of Medical Oncology, Sun Yat-Sen University Cancer Center, State Key Laboratory of Oncology in South China, Collaborative Innovation Center of Cancer Medicine, Guangzhou 510060, China

## Abstract

随着化疗药物和放射技术的进步，尤其是放化疗综合治疗的应用，经典型霍奇金淋巴瘤（cHL）患者治愈率提高，死亡率下降，但仍有部分患者复发或难治，或由于放化疗相关不良反应而死亡。近些年来随着对cHL研究的深入，治疗已经全面进入靶向免疫时代，以靶向CD30抗体偶联药物以及免疫检查点抑制剂为代表的新药进一步改善了初诊和复发/难治cHL患者的预后。本文旨在描述新药时代下如何实现cHL的全程优化管理，改善患者预后，提高疗效的同时减少不良反应发生。

## Abstract (en)

The cure rate of patients with classical Hodgkin's lymphoma (cHL) has significantly improved and the mortality rate has decreased with the advancement of chemotherapy and radiotherapy, especially the application of combined radio-chemotherapy. However, some patients still face relapse or refractory issues, as well as the risk of death due to treatment-related adverse reaction. Treatment has fully entered the era of targeted therapy in recent years, with the deepening research on cHL. Novel drugs, represented by targeted CD30 antibody-drug conjugates and immune checkpoint inhibitors, have further improved the prognosis of patients with newly diagnosed and relapsed/refractory cHL. This study aims to determine the mechanism of achieving comprehensive optimization management of cHL in the new era of drugs, improve patient prognosis, enhance therapeutic efficacy, and reduce the occurence of adverse reactions.

经典型霍奇金淋巴瘤（cHL）占霍奇金淋巴瘤的95％，是来源于生发中心B细胞的恶性肿瘤[1]，中国霍奇金淋巴瘤的发病率为0.57/10万[2]。过去几十年间，随着化疗药物和放疗技术的进步，尤其是放化疗综合治疗的应用，cHL患者5年生存率达80％以上[3]，但是治疗相关不良反应不容忽视[4]–[5]。近年来的研究进一步聚焦如何在确保疗效的前提下，减少治疗相关并发症。同时，对于复发/难治（R/R）的患者，如何走出治疗瓶颈，延长总生存（OS）期仍是当前研究的重点。随着多种新型靶向免疫药物的应用，cHL患者的预后将进一步得到改善。本文基于现有治疗挑战，讨论如何优化cHL治疗。

一、cHL一线治疗

cHL一线标准的治疗是依据2014版Lugano分期及有无预后不良因素进行分层治疗。Ⅰ～Ⅱ期cHL采用化疗［2～4个周期ABVD（阿霉素+博来霉素+长春花碱+达卡巴嗪）方案］联合放疗的综合治疗；Ⅲ～Ⅳ期cHL采用化疗为主的方案，包括ABVD方案及BEACOPP（博来霉素+依托泊苷+阿霉素+环磷酰胺+长春新碱+丙卡巴肼+泼尼松）方案[6]。然而现有标准治疗下仍有10％～15％的Ⅰ～Ⅱ期、15％～35％的Ⅲ～Ⅳ期患者进展为复发或难治性疾病[4]。同时放化疗相关不良反应，包括肺毒性、男性患者的生殖毒性[7]，第二肿瘤和心血管疾病对cHL患者的预后也会产生较大的影响[8]。因此，进一步提高cHL患者一线治疗的疗效，同时减轻治疗相关不良反应至关重要。

（一）中期PET-CT（iPET-CT）评估在一线治疗中的重要作用

iPET-CT一般指治疗1～3个周期后的PET-CT结果，可以评估化疗的敏感性，同时也是重要的预后指标[9]。基于iPET-CT结果，可以对治疗反应良好的患者降低后续治疗强度或减少疗程以减轻不良反应，对未达到良好反应的患者在早期进行治疗调整，在提高治愈可能性的同时，减少治疗相关不良反应的发生。对于Ⅰ～Ⅱ期cHL，RAPID研究[10]结果显示iPET-CT阴性患者接受3个周期ABVD方案联合30 Gy受累野放疗（IFRT）相比单纯ABVD方案治疗，3年无进展生存（PFS）率差异无统计学意义（94.6％对90.8％，*P*＝0.160）；另一项EORTC/LYSA/FIL H10研究[11]–[12]，10年的随访结果显示对于预后不良组iPET-CT阴性的患者，化疗联合放疗相比未行放疗，10年PFS率差异无统计学意义（91.4％对86.5％，*P*＝0.160）。

对于Ⅲ～Ⅳ期cHL，iPET-CT驱动治疗策略已在多个研究中得到验证并被临床广泛应用[13]–[16]。RATHL研究[13]根据2个周期化疗后PET-CT评估（PET-2）结果阴性或阳性将ABVD方案降阶为AVD（阿霉素+长春花碱+达卡巴嗪）方案或升阶为BEACOPP方案治疗。3年随访结果显示PET-2阴性患者AVD组与ABVD组PFS（*P*＝0.48）和OS（*P*＝0.76）相似，表明早期完全代谢缓解（CMR）的患者后续可以免除博来霉素治疗，PET-2阳性的患者接受增强方案治疗的3年PFS率显著高于回顾性研究中继续接受ABVD治疗的患者[13],[17]。在美国西南肿瘤协作组（SWOG）S0816研究中观察到相同的结果，PET-2阳性患者ABVD升阶为escBEACOPP增强方案（剂量增强的博来霉素+依托泊苷+阿霉素+环磷酰胺+长春新碱+丙卡巴嗪+泼尼松），5年PFS率为66％，远高于预期的15％～30％[14]。AHL2011和HD18研究[15]–[16]采用了起始为escBEACOPP的治疗方案，其中AHL2011研究将PET-2/PET-4阴性患者由escBEACOPP方案降阶为ABVD方案化疗，中位随访67.2个月，两组患者的5年PFS率差异无统计学意义，PET-2/PET-4阴性降阶治疗后患者的5年PFS率可达90.5％。在德国霍奇金研究小组（GHSG）HD18研究[16]中，PET-2阴性患者缩短了escBEACOPP的化疗周期，以降低化疗相关不良反应发生率，但对疗效无影响，5年PFS率达到91％。然而PET-CT评估仍存在一定的局限性，RATHL、SWOG S0816研究虽然证明了PET-CT评估有助于治疗策略的调整，但其中仍有15％～24％的PET阴性患者复发[13]–[14]。因此，新的预后标志物或纳入包括PET-CT指标在内的多种因素的预后模型以细化cHL患者的风险分层是未来研究的方向。其次，考虑到escBEACOPP方案的近期和远期不良反应以及患者的耐受性，其在我国临床实践中尚未得到广泛使用。对于iPET-CT阳性的患者，亟需可提高疗效、潜在毒性较小的替代方案。

（二）免疫靶向药物优化cHL一线治疗

1. 维布妥昔单抗（brentuximab vedotin，BV）：Ⅰ～Ⅱ期cHL：Kumar等[18]的研究纳入了117例早期预后不良的患者，包含下述危险因素之一：纵隔肿块（长径≥10 cm或纵隔肿块最大径/胸腔最大径>1/3）；红细胞沉降率≥50 mm/h，或红细胞沉降率≥30 mm/h合并B症状；结外累及；>2个淋巴结部位受累（根据GHSG定义）；膈下疾病。接受4个周期BV+AVD方案化疗后，PET-4阴性患者在后续接受不同剂量的放疗，分别为30 Gy受累部位放疗（involved site radiotherapy，ISRT）（队列1）、20 Gy ISRT（队列2）、30 Gy巩固放疗（CVRT，放疗部位缩小、剂量不变）（队列3）以及无放疗（队列4）。队列1至4的2年PFS率分别为93％、97％、90％和97％，研究结果提示BV的早期应用有利于降低放疗剂量甚至使患者免于放疗[18]。BREACH研究[19]进一步比较BV+AVD方案（4个周期BV+AVD，序贯放疗）和ABVD方案（4个周期ABVD，序贯放疗）的疗效，BV+AVD组2年PFS率高于ABVD组（97.3％对92.6％）。

Ⅲ～Ⅳ期cHL：ECHELON-1研究[20]经过7年长期随访，证实接受BV+AVD方案治疗患者相比ABVD方案治疗的患者：7年PFS率显著提高，进展或死亡风险降低32％；7年OS率显著提高，死亡风险降低38％，PET-2阴性和阳性亚组患者中，BV+AVD组较ABVD组均有更高的7年OS率（95.0％对90.2％，90.7％对74.0％）。安全性方面，BV+AVD组肺毒性、不孕以及第二肿瘤发生率均低于ABVD组[20]，发生周围神经病变的患者中有86％的患者随着时间的延长，其症状获得改善或缓解。在初治晚期cHL患者中BV+AVD是首个较ABVD显示OS改善的方案[21]。另一项Ⅲ期研究（GHSG HD21）评估了BV改良的escBEACOPP方案在cHL一线治疗中的有效性及安全性，研究结果表明BrECADD（BV+依托泊苷+环磷酰胺+阿霉素+达卡巴嗪+地塞米松）方案相比escBEACOPP方案可获得更高的4年PFS率（94.3％对90.9％，*P*＝0.035），且BrECADD方案能够显著降低治疗相关不良事件的发生率[22]–[24]，有望成为60岁以下晚期患者中更优的治疗选择。

2. 程序性死亡受体1（programmed cell death protein 1，PD-1）单抗：针对Ⅰ～Ⅱ期cHL早期患者，在Ⅱ期NIVAHL研究[25]中，纳入109例患者，纳武利尤单抗（Nivolumab，Nivo）联合或序贯AVD方案治疗早期预后不良患者，所有患者在化疗结束后均接受30 Gy ISRT，治疗结束时，完全缓解（CR）率为100％。中位随访41个月，联合组和序贯组的3年OS率均为100％，3年PFS率分别为100％和98％[25]。Allen等[26]的研究报道了早期预后不良患者接受3个周期帕博利珠单抗（Pembrolizumab，Pembro）后再接受4～6个周期AVD方案化疗，患者均未接受放疗。该研究除纳入12例早期预后不良患者外，也纳入了18例Ⅲ～Ⅳ期患者，中位随访时间为33.1个月，33个月PFS率保持在100％。针对Ⅲ～Ⅳ期初诊cHL晚期患者，在CheckMate 205 Ⅱ期研究[27]的队列D中，使用Nivo单药序贯Nivo+AVD方案，治疗结束时，客观缓解率（ORR）为84.0％、CR率为67.0％、9个月PFS率为92.0％。

基于上述研究结果，近期一项随机、对照、Ⅲ期研究（SWOG S1826研究[28]）进一步对BV+AVD方案及Nivo+AVD方案一线治疗cHL的疗效与安全性进行了对比，研究共纳入976例Ⅲ～Ⅳ期患者，1年的随访结果显示Nivo+AVD方案相比BV+AVD方案可获得更高的1年PFS率（94.0％对86.0％）。但该研究随访时间较短，后续疗效需要长期随访数据进一步明确。

除了对两种不同机制的新药进行对比，研究者们也在探索通过新药联合进一步减少化疗药物的种类或剂量。SGN35-027是一项正在进行的开放标签、多中心Ⅱ期临床试验，旨在评估BV+Nivo+AD（阿霉素+达卡巴嗪）方案治疗早期以及进展期cHL的疗效，其中Part B纳入了Ⅱ期（纵隔肿块长径≥10 cm）、Ⅲ期或Ⅳ期cHL患者，ORR为95.0％、CR率达到89.0％、18个月的PFS率为93.0％；Part C纳入了无大纵隔肿块（长径<10 cm）的Ⅰ期或Ⅱ期cHL患者，ORR为98.0％、CR率为93.0％，尚无PFS结果[29]–[30]。安全性方面，3级以上不良反应发生率为35％。常见的不良反应为恶心（66％）、周围神经病变（42％）和疲劳（38％）。常见的免疫相关不良反应为甲状腺功能亢进（6％）和甲状腺功能减退（6％）。该研究结果初步表明新药联合可以进一步提高疗效。NCT03331341 Ⅱ期研究[31]中，使用Pembro+AVD方案，患者2年PFS率和OS率分别为97.0％和100％，安全性方面≥3级非血液学不良反应发生率为40.0％。

二、R/R cHL治疗

R/R cHL患者首选二线挽救方案化疗后行大剂量化疗（high-dose chemotherapy，HDC）联合auto-HSCT，但是仅50％的患者可获得治愈[32]。移植前尽可能达到CR以及移植后针对高复发风险的患者进行巩固治疗有利于降低复发风险。

（一）移植前R/R cHL的治疗选择

传统化疗方案治疗R/R cHL患者的CR率为10％～41％，靶向免疫治疗的加入使R/R cHL患者获得更高的CR率（67％～95％）。

Moskowitz等[33]的研究显示复发患者接受2个周期BV单药治疗后，27％PET阴性（DS：1～2分）的患者接受了HDC或auto-HSCT治疗，其余PET阳性的患者接受2个周期增强ICE（异环磷酰胺+卡铂+依托泊苷）方案后进行HDC或auto-HSCT，最终76％的患者达到CMR。后续多项研究进一步证实了BV联合不同化疗方案这一优化理念，例如联合ICE、ESHAP（依托泊苷+甲泼尼龙+阿糖胞苷+顺铂）、DHAP（顺铂+地塞米松+阿糖胞苷）、Nivo等可以获得较高的CR率（61％～81％）[34]–[39]。除BV外，PD-1单抗联合传统化疗方案，例如Pembro联合GVD（吉西他滨+长春瑞滨+脂质体阿霉素）、Nivo联合ICE等，也可以提升R/R cHL患者的CR率（71％～95％）[40]–[42]。尽管目前尚无随机对照研究明确最佳二线挽救方案，但新药的引入已经改变了以往的治疗模式。近期一项多中心回顾性研究比较了移植前挽救治疗方案的疗效，被纳入的方案包括传统化疗、BV联合化疗、BV单药以及含免疫检查点抑制剂（CPI）方案。结果显示，含CPI方案（共65例，其中61例接受BV+CPI治疗）的2年无事件生存（EFS）率高于其他方案（*P*<0.0001）。BV+CPI方案的2年PFS率高于传统化疗（98.0％对68.8％，*P*<0.0001），提示新药联合方案可有效改善R/R cHL患者的长期生存[43]，详细数据见表1。

**表1:** 复发/难治经典霍奇金淋巴瘤（R/R cHL）患者移植前治疗方案相关研究

文献来源	治疗方案	研究设计	R/R cHL（例）	ORR（％）	CR率（％）	auto-HSCT比例（％）	PFS率（％）	OS率（％）
Moskowitz等[33]	BV后行PET，PET 阳性：augICE	Ⅱ期	45	96.0	76.0	98.0	–	2年：95.0
Kersten等[34]	BV+DHAP	Ⅱ期	55	90.0	81.0	85.0	2年：74.0	2年：95.0
Garcia-Sanz等[35]	BV+ESHAP	Ⅰ～Ⅱ期	66	91.0	70.0	91.0	30个月：71.0	30个月：91.0
LaCasce等[36]	BV+苯达莫司汀	Ⅱ期	55	93.0	74.0	73.0	2年：63.0	2年：94.0
Lynch等[37]	BV+ICE	Ⅰ～Ⅱ期	43	91.0	74.0	86.0	2年：80.4	2年：97.8
Broccoli等[38]	BV+苯达莫司汀	Ⅱ期	38	84.2	CMR：78.9	86.8	1年：78.9 3年：67.3	1年：100 3年：88.1
Cole等[39]	BV+吉西他滨	Ⅰ～Ⅱ期	42	74.0	57.0	53.0％（24/45）进行外周血干细胞采集	–	–
Moskowitz等[40]	Pembro+GVD	Ⅱ期	39	100	95.0	95.0	13.5个月：100	NA
Mei等[41]	Nivo Nivo+ICE	Ⅱ期	34 9	81.0 100	71.0 89.0	77.0	2年：72.0	2年：95.0
Advani等[44]	BV+Nivo	Ⅰ～Ⅱ期	93	85.0	67.0	90.0	3年：77.0	3年：93.0

**注** ORR：客观缓解率；CR完全缓解；PFS无进展生存；OS总生存；BV：维布妥昔单抗；PET：正电子发射计算机断层显像；augICE：增强异环磷酰胺+卡铂+依托泊苷；BV+DHAP：维布妥昔单抗+顺铂+地塞米松+阿糖胞苷；BV+ESHAP：维布妥昔单抗+依托泊苷+甲泼尼龙+阿糖胞苷+顺铂；BV+ICE：维布妥昔单抗+异环磷酰胺+卡铂+依托泊苷；Pembro+GVD：帕博利珠单抗+吉西他滨+长春瑞滨+脂质体阿霉素；Nivo：纳武利尤单抗；Nivo+ICE：纳武利尤单抗+异环磷酰胺+卡铂+依托泊苷；BV+Nivo：维布妥昔单抗+纳武利尤单抗；CMR：完全代谢缓解；–：未提及；NA：无结果

（二）移植后R/R cHL的治疗选择

AETHERA是唯一针对移植后巩固治疗的Ⅲ期研究，329例患者在auto-HSCT后1∶1随机分配，接受BV或者安慰剂进行巩固治疗，BV治疗组PFS期较安慰剂组显著延长（中位PFS期：42.9个月对24.1个月，*HR*＝0.57，95％ *CI* 0.40～0.81，*P*＝0.001）。在随访5年后，BV治疗组持续获益，5年PFS率高于安慰剂组（59％对41％），87％的安慰剂组患者接受BV作为后续治疗，可使疾病得到较好控制[45]。然而并非所有的患者都可以完成16个周期全剂量治疗，因此Wagner等[46]评估了auto-HSCT后接受不同剂量BV巩固治疗对高危患者预后的影响。研究根据接受BV总累积剂量占计划总剂量（1.8 mg/kg，16个周期）的百分比分为3组，队列1的BV总累积剂量>75％（12～16个周期）、队列2的BV总累积剂量为51％～75％（8～12个周期）、队列3的BV总累积剂量≤50％（≤8个周期）。中位随访2.96年，所有患者的2年PFS率为80.7％，队列1至3的2年PFS率分别为89.2％、86.2％、77.9％，差异无统计学意义（*P*＝0.70）。但PFS多因素分析结果显示，接受BV总累积剂量≤50％的患者，预测PFS有恶化趋势（*OR*＝1.57），auto-HSCT前对BV治疗敏感的患者可能是BV巩固治疗获益的正相关因素（*OR*＝0.19）[46]。

除BV外，以下巩固治疗药物和方案也在探索中：①Pembro作为巩固治疗的Ⅱ期临床试验显示18个月PFS率为82％、OS率为100％[47]；联合使用BV+Nivo作为巩固治疗的Ⅱ期临床试验显示18个月PFS率为94％[48]。②auto-HSCT后放疗相关研究显示，放疗可显著提高患者的2年PFS率（67％对42％，*P*<0.01）[49]。但以上均为小样本研究，仍需进一步验证。

（三）不符合移植条件的R/R cHL的治疗

不符合移植条件的R/R cHL患者预后较差，接受传统化疗，中位OS期仅为1～2年。患者多为年龄>60岁、器官功能障碍或存在合并症，还有一部分患者对HDC耐受性差[50]。此类患者群体的治疗目标是加深和维持疾病缓解状态，并且最大程度控制治疗期间药物相关不良反应以维持良好的生活质量。尽管有研究显示BV联合方案及PD-1单抗可作为此类患者的治疗选择，但仍缺乏基于明确研究数据的共识性方案。BV联合苯达莫司汀治疗R/R cHL患者的1/2期研究，中位随访23个月，非移植队列（13例）的ORR为85％，2年PFS率和OS率分别为63％和94％，安全性数据提示该方案耐受性良好[36]。Ⅱ期KEYNOTE-087研究中队列2纳入81例使用Pembro治疗的不符合移植条件的R/R cHL患者，中位随访63.7个月，该队列ORR达64.2％，中位PFS期为11.1个月[51]。以上提示CPI和BV可为不符合移植条件的患者带来生存获益，同时，相比于传统化疗方案，患者耐受性更好。

（四）auto-HSCT后复发或多线复发的R/R cHL的治疗

既往研究显示auto-HSCT后出现疾病进展的cHL患者中位OS期仅为17～29个月，原发难治或不适合移植患者的OS期相对较短[52]。目前针对这类患者开展的BV和PD-1单抗单药研究展示了良好的疗效[51],[53]–[55]。Ⅲ期KEYNOTE-204研究比较了Pembro和BV在R/R cHL患者中的疗效，结果显示Pembro治疗的中位PFS期长于BV，但该研究中两组既往中位治疗线数存在差异（Pembro组为2，BV组为3），治疗周期也不同[56]，Pembro组接受了15个周期的治疗，BV组仅接受7个周期治疗。除单药治疗外，Nivo联合苯达莫司汀治疗的CR率可达57％[57]、卡瑞利珠单抗联合地西他滨治疗的CR率可达71％[58]，为单药治疗不能满足治疗需求的患者提供了选择。BV初始治疗获得缓解后复发的R/R cHL患者接受BV再治疗仍可再次获益，Bartlett等[59]的研究显示BV再治疗在R/R cHL患者中的ORR为60％、CR率为30％。最新的美国真实世界数据显示，6 442例使用BV初始治疗的cHL患者中，有13％接受了BV再治疗，结合临床研究所报道的BV再治疗ORR为53％～60％，提示BV再治疗可成为这类患者的治疗选择之一[60]。

除药物治疗外，如果此类患者身体状况良好，接受挽救治疗获得部分缓解，则可以尝试allo-HSCT[61]，经allo-HSCT治疗后的5年OS率为30％～50％[62]，但最佳的预处理方案和移植后急性移植物抗宿主病（acute graft-versus-host disease，aGVHD）的预防措施尚无定论。采用清髓性预处理方案的allo-HSCT患者非复发死亡率（non-relapse mortality，NRM）较高，减低毒性预处理方案可以降低NRM[63]。移植后输注环磷酰胺可以预防aGVHD[64]，但仍需更多数据予以验证。Simeunovic等[65]回顾性分析了62例移植后复发或多线复发的R/R cHL患者，其中77％采用减低毒性预处理方案，接受allo-HSCT后，中位随访61个月，2年和5年OS率分别为54％和50.2％、2年和5年NRM分别为23.1％和27.4％。Beynarovich等[66]的回顾性研究纳入了58例移植后复发或多线复发的R/R cHL患者，该研究采用FluBe（氟达拉滨+苯达莫司汀）预处理方案联合移植后输注环磷酰胺，allo-HSCT后中位随访20个月，3年OS率为81％。BV和CPI在allo-HSCT前使用可以增加患者的化疗敏感性从而提高生存率。Faisal等[67]的研究将行allo-HSCT的R/R cHL患者按接受治疗的时间分为1代组（2001年至2010年）和2代组（2011年至2017年），1代组患者无BV/CPI治疗史，2代组中95％患者有BV治疗史，结果显示两组的4年OS率分别为34％和83％（*P*<0.001）、4年PFS率分别为28％和62％（*P*＝0.001）。以上提示allo-HSCT可作为移植后复发或多线复发的R/R cHL患者的治疗选择，但最佳移植前预处理方案仍需探索，随着BV等新型药物的出现，R/R cHL患者的治疗方案实施顺序正在不断优化，引入allo-HSCT的最佳时机尚未确定[67]。此外，对于allo-HSCT前使用CPI的R/R cHL患者，需考虑潜在的免疫相关不良反应发生风险增加[68]。

目前多种新型药物的研究正在开展。Ramos等的Ⅰ/Ⅱ期研究（NCT02690545、NCT02917083）评估了CD30 CAR-T细胞治疗多线治疗耐药的R/R cHL患者的疗效，该研究纳入了41例R/R cHL患者，中位治疗线数为7，中位随访时间533 d，37例疗效可评估患者的1年PFS率和OS率分别为36％和94％[69]。CD30/CD16A双特异性抗体（AFM13），通过结合并激活自然杀伤细胞和巨噬细胞，从而诱导对CD30阳性肿瘤细胞的特异性和选择性杀伤，AFM13联合Pembro治疗R/R cHL患者的Ⅰb期研究结果提示接受该方案治疗的患者ORR可达83％[70]–[71]。淋巴细胞激活基因-3（LAG-3）抗体联合PD-1单抗复合制剂治疗R/R cHL的Ⅰ/Ⅱ期研究显示，该联合方案治疗的15个月PFS率为33％～53％、15个月OS率为87％～93％[72]，该联合方案对比传统化疗的Ⅲ期随机对照研究（Keyform-008）也正在开展[73]，目前尚无数据。另外，我国自主研发的抗BTLA单克隆抗体（JS004）联合PD-1单抗（特瑞普利单抗）复合制剂治疗PD-1单抗耐药的cHL的Ⅲ期临床研究也在进行中[74]。靶向CD25的抗体偶联药物（Camidanlumab Tesirine，Cami）的Ⅱ期研究结果显示Cami治疗多线治疗耐药（中位治疗线数为6）的R/R cHL患者的ORR可达70.1％、CR率为33.3％，中位随访时间10.7个月，中位PFS期为9.1个月[75]，但其治疗相关不良反应导致开发中止。新药疗效和安全性的不断探索可能为使用BV或PD-1治疗后疾病进展的患者带来希望。

三、结论与展望

cHL治疗优化的目标是在确保疗效的前提下，减少治疗相关并发症，改善预后，延长OS期。在cHL一线治疗中，iPET-CT评估对患者有重要意义，可以根据患者对治疗的反应调整后续的治疗策略。在原有传统化疗方案的基础上联合靶向免疫治疗进行优化，可以使cHL患者一线治疗的治愈率进一步提升。对于R/R cHL的患者，使用挽救治疗联合新型靶向免疫治疗可以获得更高的CR率，让更多的患者有机会接受auto-HSCT，同时对高危复发风险患者采用新药进行巩固治疗有利于降低复发风险。对于BV或PD-1单抗复发难治的患者，CD30 CAR-T细胞、LAG-3抗体联合PD-1单抗复合制剂以及CD25单抗等新药的研发和转化可能为cHL患者带来新的选择和治愈的希望。
